# Burkholderia cepacia Infective Endocarditis of Native Aortic Valve: A Case Report and Review of Literature

**DOI:** 10.7759/cureus.71548

**Published:** 2024-10-15

**Authors:** Isa Almubarak, Abdulla J Almubarak, Yusuf A Ahmed, Manar A Ali, Walaa H Yusuf, Mariam Ismail, Shady Elhadidi, Hanaa Abdelaziz, Mohamed A Gabr, Gehad Awad

**Affiliations:** 1 Department of General Surgery, Al Kindi Hospital, Manama, BHR; 2 Faculty of Medicine, Mansoura University, Mansoura, EGY; 3 Department of General Surgery, Dammam Medical Complex, Dammam, SAU; 4 Department of Cardiothoracic Surgery, Delta University for Science and Technology, Mansoura, EGY; 5 Department of Cardiothoracic Surgery, Faculty of Medicine, Mansoura University, Mansoura, EGY; 6 Department of Cardiovascular Medicine, Faculty of Medicine, Mansoura University, Mansoura, EGY

**Keywords:** aortic valve mass, aortic valve replacement, burkholderia cepacia, case report, infective endocarditis

## Abstract

Infective endocarditis (IE) is a serious cardiac infection of the endocardium, native and prosthetic valves, or cardiac device. In this case study, we report a case of an immunocompetent patient with severe *Burkholderia cepacia* aortic valve endocarditis.

A 54-year-old female presented to the emergency department with progressive shortness of breath, chest pain, palpitations, and cough for a period of 20 days. On physical examination, the patient was orthopneic, tachypneic, and tachycardic with an irregularly irregular rhythm. Her blood pressure was 110/80, with an oxygen saturation of 88% on room air. On auscultation, variable S1 intensity, weak S2, ejection systolic murmurs all over the precordium, and bilateral crepitations were heard over lung bases. Electrocardiography was performed, which showed atrial fibrillation with rapid ventricular response. Transthoracic echocardiography and transesophageal echocardiography were performed, which revealed a large aortic valve mass causing severe valvular obstruction. Blood culture results were non-conclusive. Autoimmune laboratory workup was conducted to exclude systemic lupus erythematosus and antiphospholipid syndrome. The patient received loop diuretics and empirical antibiotics initially, and an urgent surgical aortic valve replacement was performed. *Burkholderia cepacia* was detected by microbiological analysis of the excised valve. Amoxicillin/clavulanic acid was given for a period of four weeks post-operatively.

*Burkholderia cepacia* could be one of the causative organisms causing IE and can affect the aortic valve in immunocompetent patients.

## Introduction

Infective endocarditis (IE) is a serious cardiac infection of the endocardium, native and prosthetic valves, or cardiac device with an annual incidence of approximately 3 to 10 cases per 100,000 individuals [1]. Globally, IE remains highly fatal, with an overall mortality rate of approximately 25% [1]. Risk factors include old age, drug abuse, valvular disease, cardiac surgery, poor dentition, and immunocompromised status [2]. Native-valve IE is rare, with an incidence ranging from 2 to 10 cases per 100,000 persons-years, predominantly affecting males with a male-to-female ratio of nearly 2:1, and diagnosed at a mean age exceeding 65 to 75 years [3]. However, younger individuals in countries with a high prevalence of rheumatic heart disease and poor health infrastructure are also susceptible [4]. There are many causative pathogens leading to the development of IE in the setting of native valves, with the majority of the cases occurring due to gram-positive *Streptococci, Staphylococci, *and* Enterococci* infection. Altogether, these three species account for 80% to 90% of all cases [5]. Another, less commonly attributed pathogens to the development of this condition are various Streptococci species. *Burkholderia cepacia* bacteria is an exceedingly uncommon causative organism of IE, with only 15 cases reported to date in the English literature (less than 20) [6]. *Burkholderia cepacia *is a gram-negative bacterium with multi-drug resistance to antibiotics such as β-lactam antibiotics, fluoroquinolones, and trimethoprim, and disinfectants [7,8]. It has been observed that this bacterium can lead to IE in individuals who are immunodeficient, abuse intravenous drugs such as heroin, and have undergone prosthetic valve replacement [9].

Here, we report a case of an immunocompetent patient with severe aortic valve endocarditis caused by *Burkholderia cepacia* requiring surgical valve replacement.

## Case presentation

A 54-year-old female presented to the emergency department with progressive shortness of breath, chest pain, palpitations, and cough for a period of 20 days. The patient has a history of hypertension and is currently on an angiotensin-converting enzyme inhibitor (ACEI), with no other significant past medical history. She has two daughters diagnosed with systemic lupus erythematosus (SLE) and a son who died of sudden cardiac death.

**Figure 1:**
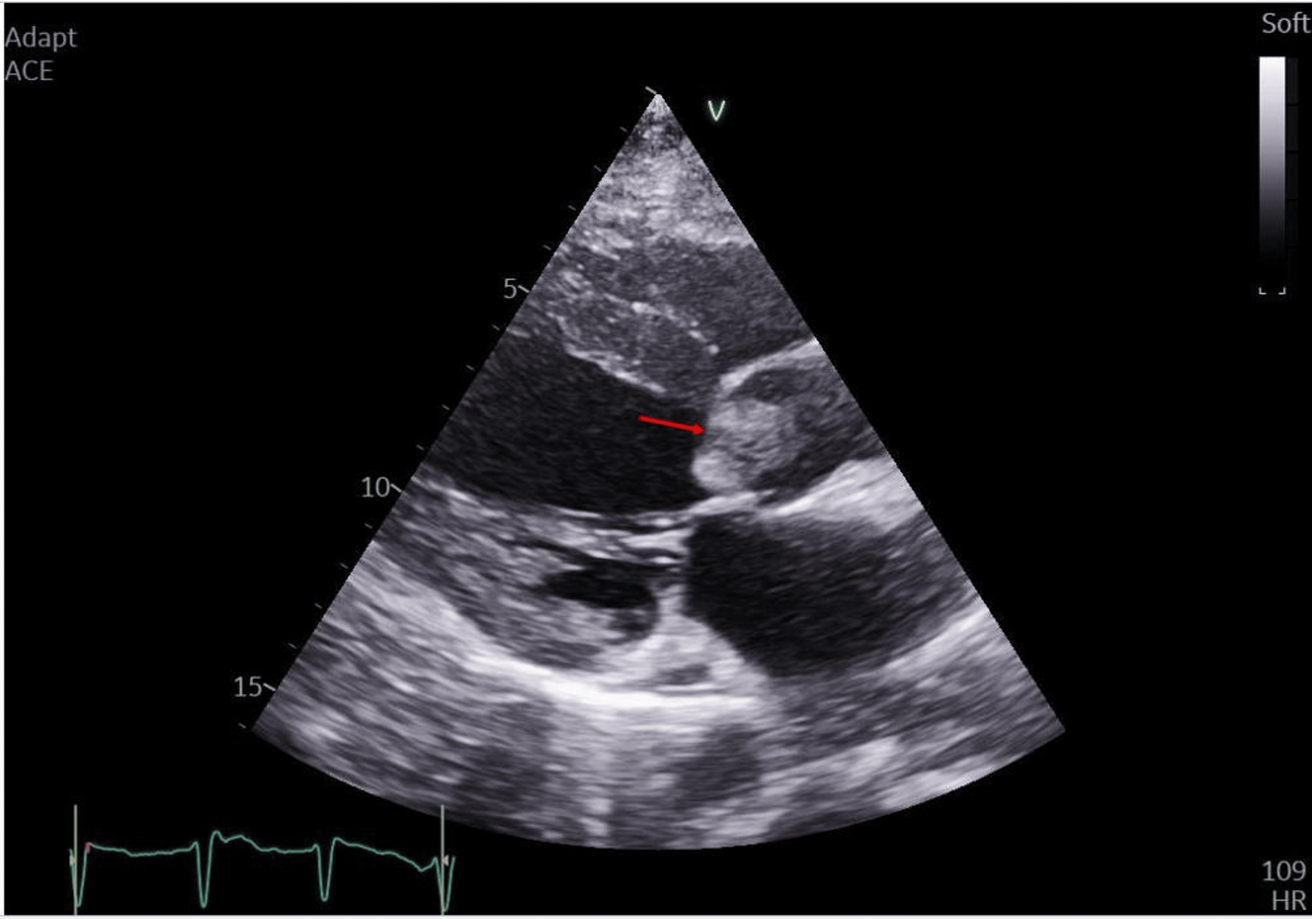
Transthoracic echocardiography (parasternal long axis view) showing the aortic valve mass measuring 2.3 x 1.4 cm (red arrow).

**Figure 2:**
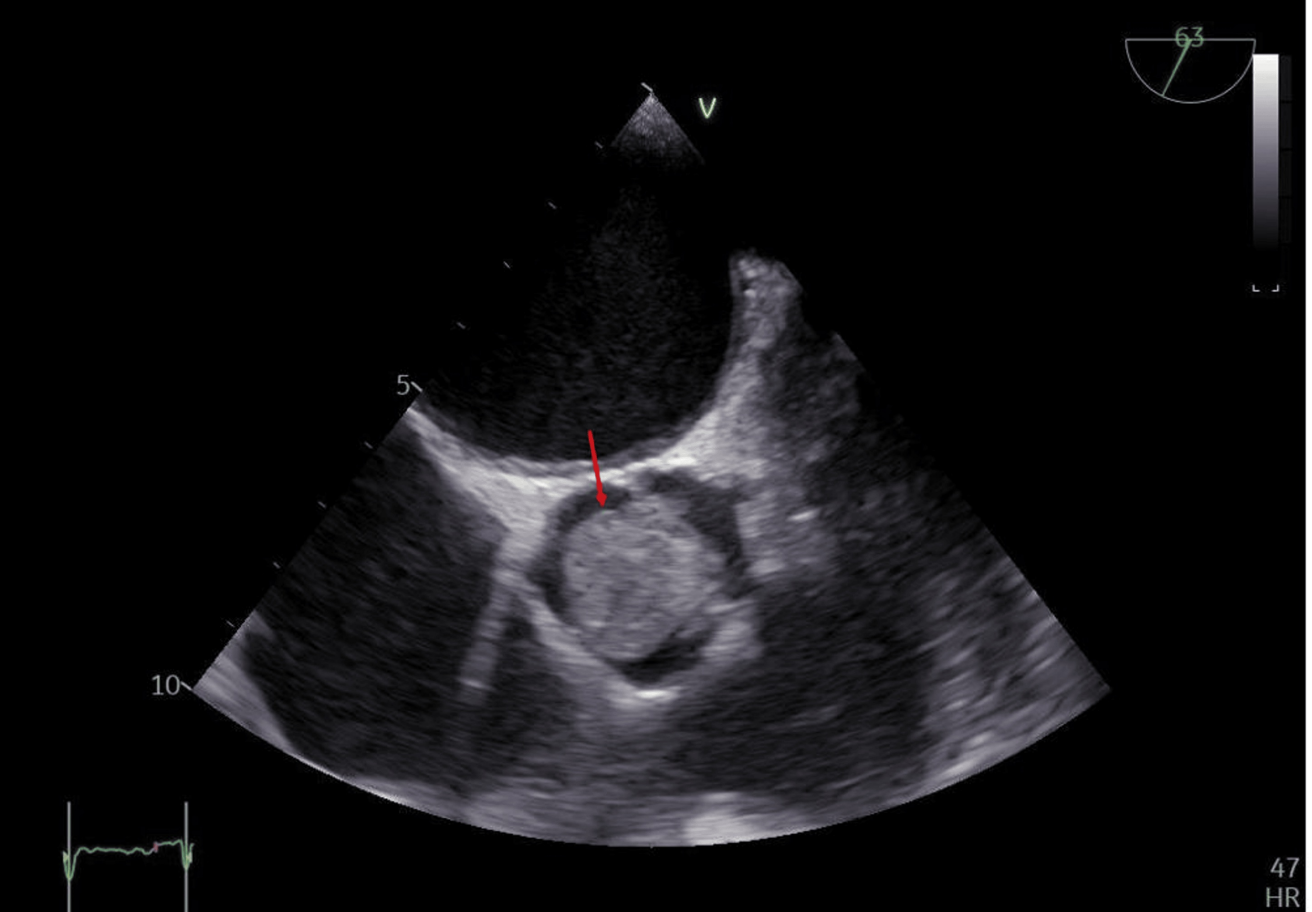
Transesophageal echocardiography (mid-esophageal short axis view) showing the aortic valve mass obstructing the aortic valve orifice, measuring 3.0 x 2.1 cm (red arrow).

Upon physical examination, the patient was feverish (temperature: 38°C), orthopneic, tachypneic, and tachycardic with an irregularly irregular rhythm. Her blood pressure was 110/80 with an oxygen saturation of 88% on room air. On auscultation, variable S1 intensity, weak S2, ejection systolic murmurs all over the precordium, and bilateral crepitations were heard over lung bases. Electrocardiogram (ECG) revealed atrial fibrillation (AF) with rapid ventricular response with no ST or T wave changes. Transthoracic echocardiography (TTE) revealed a large aortic valve mass, measuring 2.3 x 1.4 cm (Figure 1), causing severe valvular obstruction with a peak gradient of 104 mmHg and a mean gradient of 71.45 mmHg. The mitral valve leaflets were sclerotic with moderate-to-severe regurgitation; the left ventricular systolic function was impaired with an ejection fraction of 33%. Transesophageal echocardiography revealed an aortic valve mass measuring 3.0 x 2.1 cm (Figure 2). Blood culture results were non-conclusive. Tumor, valve myxoma, and marantic endocarditis were also considered in the differential diagnosis. Autoimmune laboratory workup was negative for SLE and antiphospholipid. The patient received IV ceftriaxone 2 mg twice a day, IV meropenem 2 mg thrice a day empirically for seven days before the operation, and IV furosemide 20 mg twice a day for two days at presentation, torosemide 20 mg once daily after stopping IV furosemide, and spironolactone 25 mg tab for three days before the operation. Cardiothoracic surgery was consulted for possible surgical management. Duration from cardiothoracic surgery consultation till surgery was three days due to interruption of oral anticoagulant (apixaban 5 mg twice a day). Then the patient underwent surgical aortic valve replacement. During operation, bicaval cannulation was performed due to interception of the mitral valve cardiopulmonary bypass time of 80 minutes and cross-clamp time of 50 minutes. Mitral valve leaflet was normal and well coapted, left atrium size was within normal range, and intra-operative TEE confirmed a mild degree of mitral regurgitation only.

**Figure 3:**
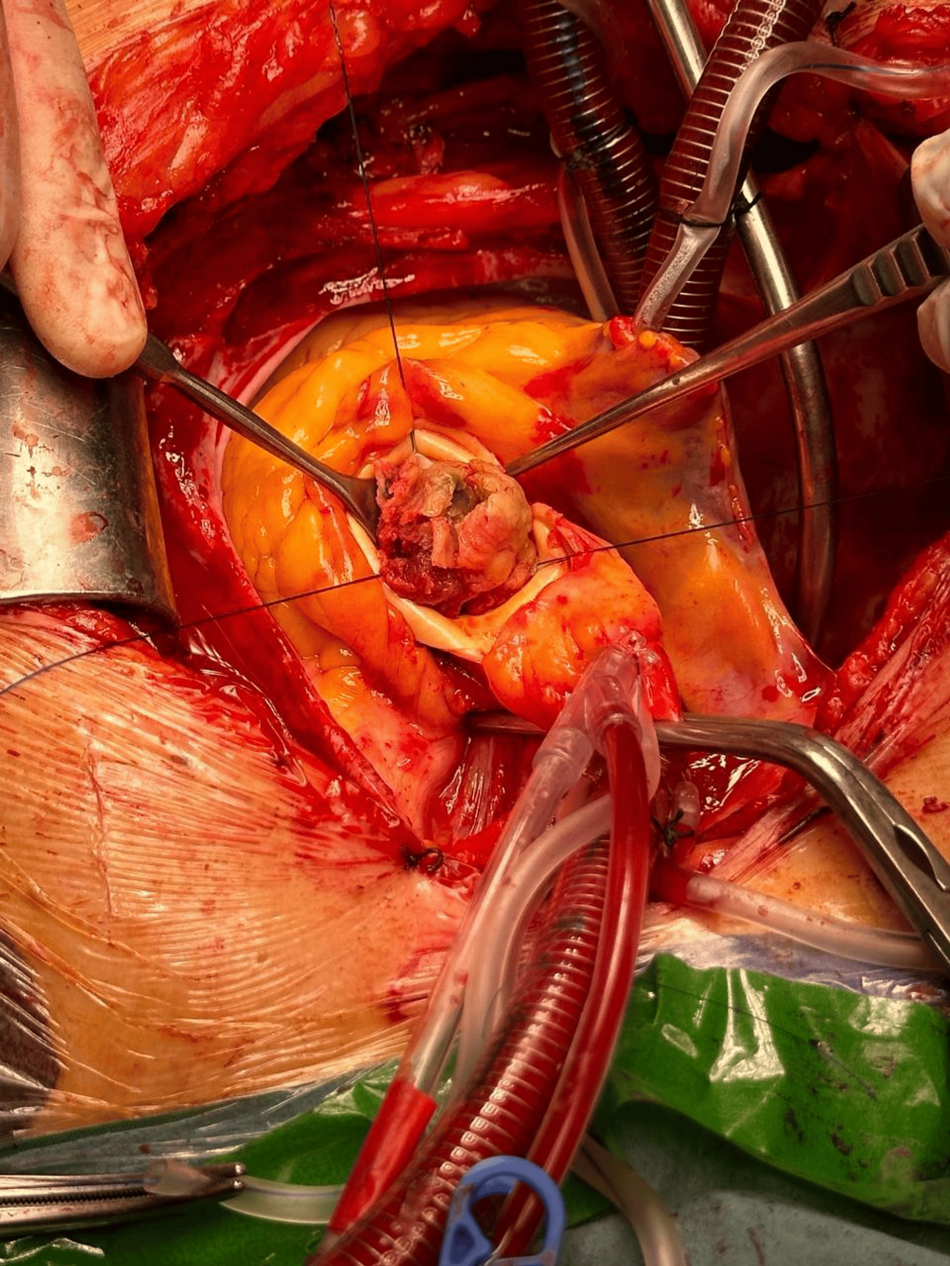
Intra-operative view of the aortic valve with a firm brownish mass measuring 3 x 3 x 2 cm protruding from it.

**Figure 4:**
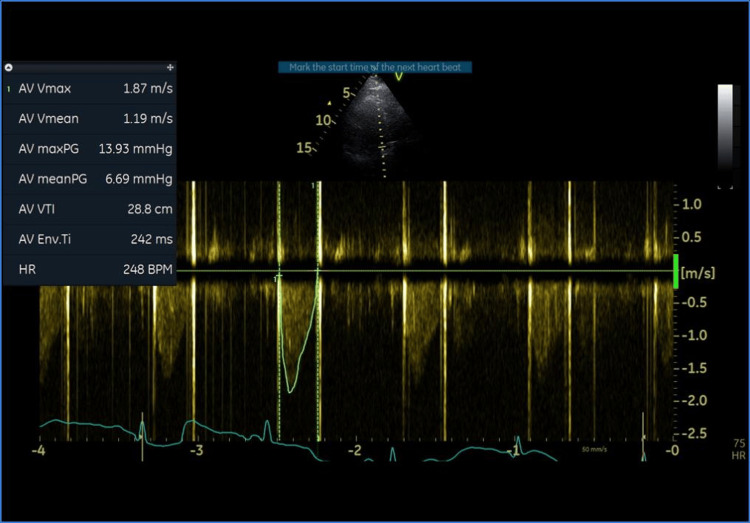
Post-operative transthoracic echocardiogram of aortic valve prosthesis with a mean gradient of 6 mmHg.

**Figure 5:**
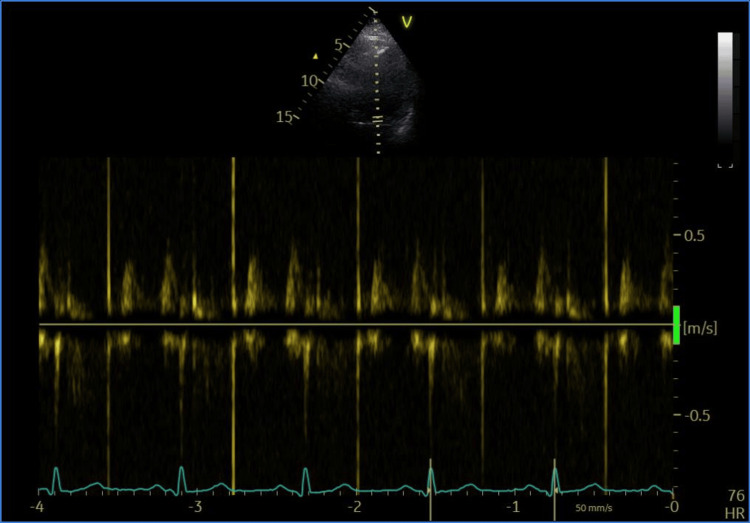
Post-operative transthoracic echocardiogram of the mitral valve.

Grossly, the mass was firm brownish with yellowish foci measuring 3 x 3 x 2 cm (Figure 3). Pathological examination showed fibrous valve tissue with scattered inflammatory cells, calcification, focal degenerative changes, and thrombi. Microbiological analysis of the excised damaged valve detected *Burkholderia cepacia*, which was sensitive to multiple antibiotics, including amoxicillin/clavulanic acid. The patient stayed in the ICU for three days and in the hospital for eight days. The duration of mechanical ventilation post-operatively was 5 hours, and she did not receive anything other than dobutamine as an inotrope, and the AF did not resolve directly after surgery but was controlled without the need of amiodarone. Amoxicillin/clavulanic acid was given for a period of four weeks. Seven days post-operatively, TTE showed a well-functioning aortic valve prosthesis with no masses, vegetation, or thrombi. Mean gradient was 5 mmHg. Six months post-operative TTE showed the mean gradient to be 6 mmHg, with no masses, vegetation, or thrombi, and good LV systolic function ejection fraction of 60% by biplane Simpson’s method (Figure 4). The patient showed good functional capacity post-operatively (New York Heart Association [NYHA] class I), with ECG showing sinus rhythm. Mitral valve was normal in thickness, with good coaptation no regurgitation (Figure 5). We can explain this as in near total obstruction of aortic valve by this huge mass or critical AS, mitral regurgitation often occurs due to secondary effects caused by LV pressure overload, dilation, or papillary muscle dysfunction, even when the mitral valve leaflets are structurally normal. This is a functional mitral regurgitation resulting from altered hemodynamics rather than a primary mitral valve disease. This theory was confirmed with the disappearance of this mitral regurgitation intra-operatively during intra-operative TEE and post-operative echocardiography.

## Discussion

In 1949, Walter Burkholder first found *Burkholderia cepacia* as the reason behind onion skin rot, while it was first identified as a human pathogen in the 1950s. In 1977, the organism was isolated from patients with cystic fibrosis. Hence, people started calling it *Pseudomonas cepacia* [10]. In mammal cells, it was elucidated that when *Burkholderia cepacia* enters the body, the amoeba forms vacuoles to contain it; the development of the amoeba is then halted, and the bacterium remains alive and active while evading amoeba digestion. Amoeba is also present in humans, especially in the nasal mucosa. The Trojan horse model has theorized that the bacterium enters the amoeba in the nasal mucosa and uses it to reach the lower respiratory tract. It was also reported that *Burkholderia cepacia* can survive in phagocytic cells such as macrophages, monocytes, and type II pneumocytes [11]. This bacterium can cause tumor necrosis factor (TNF)-mediated inflammation or bacteremia, causing, in rare cases, sepsis-like manifestation and endocarditis [12]. In our patient, it was speculated that the cardiac involvement was due to a previous respiratory infection two to three weeks before presentation with fever and cough. We except that the mass increased in size progressively with the beginning of the patient's symptoms as she was previously asymptomatic.

*Burkholderia cepacia* is a catalase-positive, aerobic, lactose-producing, gram-negative bacillus that commonly affects patients with immunodeficiency, with specific predilection to patients with cystic fibrosis and chronic granulomatous disease. Intravenous drug users are also at risk of infection with this pathogen [10]. One factor that makes this case unique is that *Burkholderia cepacia *infected the patient without the presence of typical predisposing factors. Also, the aortic valve was infected instead of the tricuspid valve, which is the most involved among infected native valves by this organism [13]. To our knowledge in English-language literature in PubMed, only three cases were reported, in which a native aortic valve was involved with the need for surgical intervention (Table 1).

**Table 1:** Summary of the cases of native aortic valve IE caused by Burkholderia cepacia that required surgical management.

Author	Year	Affected valves	Age	Gender	Main symptoms	Medical management	Surgical intervention	Outcome
Hamilton et al. [14]	1973	Aortic valve	29 years	Male	SOB	Chloramphenicol, trimethoprim/sulfamethoxazole	Aortic valve replacement	Improved
Khan et al. [13]	2017	Aortic valve	44 years	Male	SOB, fever, chills, fatigue	Ciprofloxacin, piperacillin/tazobactam, vancomycin	Aortic valve replacement	Improved
Sabir et al. [15]	2018	Aortic and mitral valves	30 years	Male	Fever, SOB	Trimethoprim-sulfamethoxazole, meropenem, voriconazole	Aortic and mitral valve replacement	Improved
Current study	2024	Aortic Valve	54 years	Female	SOB, chest pain, palpitation, cough	Amoxicillin/clavulanic acid	Aortic valve replacement	Improved

According to the 2023 European Society of Cardiology (ECS) guidelines for the management of endocarditis [16], early intervention with surgery is considered in patients with severe valvular pathology, heart failure, cardiogenic shock or pulmonary edema, local complications (abscess, false aneurysm, fistula, enlarging vegetation), persistent infection, and poor hemodynamic tolerance. Our patient met the indications for surgical intervention (severe valvular pathology and heart failure with an ejection fraction of 33%, large obstructing aortic vegetation, more than 10 mm, with a risk of embolization) and thus underwent urgent aortic valve replacement. The mitral valve was not replaced due to it being healthy and functional on transesophageal echocardiography intraoperatively after replacing the aortic valve. We can explain this as in near total obstruction of aortic valve by this huge mass or critical AS, mitral regurgitation often occurs due to secondary effects caused by LV pressure overload, dilation, or papillary muscle dysfunction, even when the mitral valve leaflets are structurally normal. This is a functional mitral regurgitation resulting from altered hemodynamics rather than a primary mitral valve disease. This theory was confirmed with the disappearance of this mitral regurgitation intraoperatively during intraoperative TEE and post-operative echocardiography.

*Burkholderia cepacia* has a high degree of antimicrobial resistance, which can result in difficulty treating this pathogen. Moreover, different case reports published earlier showed variable responses to management, ranging from successfully treating it with antibiotics only without the need for surgical intervention to requiring both antibiotics and surgical intervention by replacement of the affected valve [9]. One other differential that was highly possible at the presentation was nonbacterial thrombotic endocarditis (marantic endocarditis), a condition characterized by the presence of aseptic fibrin depositions on cardiac valves due to hypercoagulability and endocardial damage [17].

## Conclusions

*Burkholderia cepacia* could be one of the causative organisms causing IE, and it can affect the aortic valve in immunocompetent patients requiring valve replacement.

## References

[1] Chen H, Zhan Y, Zhang K (2022). The Global, Regional, and National Burden and Trends of Infective Endocarditis From 1990 to 2019: results from the Global Burden of Disease Study 2019. Front Med (Lausanne).

[2] Mettler SK, Alhariri H, Okoli U (2023). Gender, age, and regional disparities in the incidence and mortality trends of infective endocarditis in the United States between 1990 and 2019. Am J Cardiol.

[3] Østergaard L, Smerup MH, Iversen K (2020). Differences in mortality in patients undergoing surgery for infective endocarditis according to age and valvular surgery. BMC Infect Dis.

[4] Mutagaywa RK, Vroon JC, Fundikira L (2022). Infective endocarditis in developing countries: an update. Front Cardiovasc Med.

[5] Yallowitz AW, Decker LC (2024). Infectious endocarditis. StatPearls [Internet].

[6] Gonzalez JM, Lowenhaar G, Ramgopal M, Chalasani P (2024). Burkholderia cepacia: a rare source of endocarditis. R I Med J (2013).

[7] Bahçeci İ, Şenol FF, Dilek AR, Yıldız İE, Arslan N, Duran ÖF (2022). Polyclonal outbreak of bacteremia caused by Burkholderia cepacia in the intensive care unit. Ann Clin Anal Med.

[8] Rhodes KA, Schweizer HP (2016). Antibiotic resistance in Burkholderia species. Drug Resist Updat.

[9] Ki HK, Kim SH, Han SW, Cheong HS (2011). A case of native valve endocarditis caused by Burkholderia cepacia without predisposing factors. BMC Infect Dis.

[10] Nnaoma C, Chika-Nwosuh O, Sossou C (2019). A rare culprit of infective endocarditis in an IV drug user: Burkholderia cepacia. Case Rep Med.

[11] Vial L, Chapalain A, Groleau MC, Déziel E (2011). The various lifestyles of the Burkholderia cepacia complex species: a tribute to adaptation. Environ Microbiol.

[12] Russo M, Nardi P, Saitto G (2017). Paravalvular leak of a mechanical mitral valve prosthesis associated with Burkholderia cepacia subacute endocarditis: a rare case successfully treated by multidisciplinary approach. Kardiochir Torakochirurgia Pol.

[13] Khan M, Lalani FK, Ikram A, Zaman G, Ahmed P (2017). Dual infection by Burkholderia Cepacia and Pseudomonas putida in an infective endocarditis case. J Coll Physicians Surg Pak.

[14] Hamilton J, Burch W, Grimmett G, Orme K, Brewer D, Frost R, Fulkerson C (1973). Successful treatment of Pseudomonas cepacia endocarditis with trimethoprim-sulfamethoxazole. Antimicrob Agents Chemother.

[15] Sabir N, Ikram A, Gardezi A, Zaman G, Satti L, Ahmed A, Khadim T (2018). Native valve dual pathogen endocarditis caused by Burkholderia cepacia and Aspergillus flavus - a case report. JMM Case Rep.

[16] Delgado V, Ajmone Marsan N, de Waha S (2023). 2023 ESC Guidelines for the management of endocarditis. Eur Heart J.

[17] Alhuarrat MA, Garg V, Borkowski P (2024). Epidemiologic and clinical characteristics of marantic endocarditis: a systematic review and meta-analysis of 416 reports. Curr Probl Cardiol.

